# How can the results of a qualitative process evaluation be applied in management, improvement and modification of a preventive community trial? The IHHP Study

**DOI:** 10.1186/0778-7367-69-9

**Published:** 2011-12-05

**Authors:** Nizal Sarrafzadegan, Katayoun Rabiei, Mousa Alavi, Heidarali Abedi, Sonia Zarfeshani

**Affiliations:** 1 Isfahan Cardiovascular Research Center, Isfahan Cardiovascualr Research Institute, Isfahan University of Medical Sciences, Isfahan, Iran; 2 Isfahan Cardiac Rehabilitation Research Center, Isfahan Cardiovascualr Research Institute, Isfahan University of Medical Sciences, Isfahan, Iran; 3 Nursing & Midwifery Care Reaserch Center, Isfahan University of Medical Sciences. Isfahan, Iran; 4 Faculty of Nursing and Midwifery, Khorasgan (Isfahan) Branch, Islamic Azad University, Isfahan, Iran; 5 Isfahan Hypertension Research Center, Isfahan Cardiovascualr Research Institute, Isfahan University of Medical Sciences, Isfahan, Iran

**Keywords:** Community interventions, Health behavior, Health, Cardiovascular, program evaluation

## Abstract

**Background:**

This study reports the results of the qualitative process evaluation (PE) of the Isfahan Healthy Heart Program (IHHP), an integrated community-based trial for prevention and control of non-communicable diseases in Iran.

**Methods:**

The study explored the overall quality of program implementation. The participants, including designers of IHHP, stakeholders and community members (n = 60) were purposefully recruited from the intervention areas. Data collected from semi-structured interviews and field notes were analyzed using a modified thematic analysis.

**Results:**

Four main themes were identified. Our findings highlighted the key role of the resources as both facilitating and hindering factors. IHHP directors faced incompatibilities arising from negative perceptions/attitudes which resulted in decreased adherence to the program. Hence various strategies were used to motivate, strengthen and organize the human workforce implementing the program.

**Conclusion:**

Recommendations arising from evaluation of the program were used in subsequent stages of implementation. Qualitative research is an important component of community trials which can improve their implementation.

## Introduction

The high prevalence of cardiovascular diseases and their associated mortality worldwide, including in Iran, have made them a major health concern [1-3]. As a public health response, a comprehensive action-oriented integrated community-based program entitled Isfahan Healthy Heart Program (IHHP) was launched in Iran in 1999-2000.

This program has provided an opportunity to assess the effectiveness of lifestyle interventions designed for non-communicable diseases (NCDs) prevention and control in a developing country setting. The long-term objectives of IHHP were to decrease the incidence of NCDs and their related disability/mortality. Improving of the knowledge and awareness of the general population and health professionals about NCDs, and improvement of the skills of health professionals in controlling NCD risk factors constituted the short-term objectives of IHHP [4].

Study communities were two intervention counties (Isfahan and Najafabad) and a reference area (Arak), all located in Central Iran. The program had three phases. The first phase, that was conducted in intervention and reference areas in 2000 to study the existing situation of cardiovascular diseases and it's risk factors as well as the knowledge, attitude and practice of the population about the disease and healthy life style. The second-phase, that included multidisciplinary interventions, began in 2001 based on the results of the situation analysis and needs assessment in the community; this phase targeted the whole community and environmental changes in Isfahan and Najafabad [4]. The third Phase was conducted in both reference and intervention areas to the evaluate outcomes by repeating the same studies done in the first phase on independent samples [5,6]. The program consisted of ten interventional projects, namely Healthy Food for Healthy Communities (HFHC), Isfahan Exercise Project (IEP), Women's Healthy Heart Project (WHHP), Heart Health Promotion from Childhood (HHPC), Youth Healthy Heart Project (YHHP), Worksite Intervention Project (WIP), Non-Governmental Organizations (NGOs) and Volunteer Intervention Project (NVIP), Health Professionals Education Project (HPEP), Healthy Lifestyles for High Risk Groups (HLHR) and Healthy Lifestyles for Cardiac Patients (HLCP). Each project targeted a different group and consisted of various strategies, including healthy nutrition, physical activity, tobacco control and stress management (Table 1) [6].

**Table 1:** Brief summary of the main activities of the ten interventional projects of Isfahan Healthy Heart Program [6]

Project name	Main interventions
Healthy Food for Healthy Community	Training about healthy cooking methods, making high-fiber low-salt bread, production of healthy food products by food industries, modifying food labels, educating the public on the concept of healthy nutrition, improving the formulation of confections, introduction of healthy brands and half-portions into restaurants and fast food shops
Isfahan Exercise and Air Pollution Control Project	Providing training on exercise and physical activity through local TV, distribution of educational CDs about exercise at home and at worksite, organizing public exercise rallies, organizing automobile-free days, organizing healthy heart exhibitions, educating about air pollution control methods through local TV, advocating the development of bicycle lanes in the city
Women Healthy Heart Project	Training the young women and their family members who attended pre-marriage classes, instructors of the Literacy Campaign Movement and their students, instructors of charities, women's Basij Movement, women in different organizations, women attending health centers and health houses, female volunteer-instructors of the Red-Crescent Society, educating the public through TV and cook books, distributing a CD about methods of physical activity requiring no special facilities
Heart Health Promotion from Childhood	Population approach by training children, parents, health professionals, school and kindergarten staff about healthy lifestyle, promotion of physical activity in schools and kindergartens, introduction of healthy snacks in schools and kindergartens and establishing healthy buffets in schools, forming role model groups from volunteer students, providing practical training through TV about healthy lifestyle; and high-risk approach by screening for CVD risk factors in children of patients with premature CVDs and children with at least one risk factor such as obesity.
Youth Intervention Project	Training volunteers from the Red Crescent Society, garrison instructors, soldiers in their mandatory military service and university students, and kitchen staff of universities and garrisons about healthy lifestyle, conducting the international anti-smoking Quit & Win campaign
Worksite Intervention Project	Training occupational medicine physicians or health assistants, introducing dietary modifications into restaurants of factories, enforcing no-smoking regulations at worksite, using the existing screening system to detect high-risk groups, encouraging physical activity at worksite, publishing health messages about CVD prevention as an integrated part of official newsletters of different organizations.
Non-Governmental Organizations and Volunteers Project	Training health workers in cities and villages, forming, training and empowerment of an assembly of health volunteers, training of the community about performing physical activity in the absence of facilities, healthy nutrition and coping with stress via trained volunteers and health-related NGOs.
Health Professionals Education Project	Establishment of educational assemblies, training GPs through continuing medical education (CME) courses, training physicians through periodical seminars, training nurses by organizing educational assemblies, and publishing and distributing books and newsletters among nurses and other health professionals in urban and rural areas, running information campaigns on various occasions
Healthy Lifestyle for High-Risk Groups	Training the personnel of the health system, high-risk individuals, retired employees, activation of clinics at hospitals, public training, distributing educational brochures to those attending pharmacies, printing health messages on laboratory report sheets
Healthy Lifestyle for Cardiovascular Patients Project	Training the patients and their families at the time of discharge, printing cards for patients to record all necessary information related to their condition, establishing rehabilitation units in all heart hospitals, improving nutrition-related and cooking procedures at hospital restaurants, distributing educational folders containing educational materials on cardiovascular disease secondary prevention and rehabilitation

IHHP evaluation protocol targeted the impact, process and outcome of the program with work beginning in 2002 [5]. We reported the results and describe the methods of the qualitative component of IHHP process evaluation (PE) [7]. Appreciating the value of process evaluation [8,9], IHHP directors set out to gain in-depth understanding of how the program played out in action. Details of process evaluation, designed as a triangular study have been reported previously [7]. Unlike most evaluation studies focusing primarily on objective and subjective outcome evaluation [8,9], we aimed to understand the quality of program implementation and explore the perceptions/experiences of program implementers/targets during the interventions. We focused on obtaining information that could help with program management and improvement.

## Methods

### Design

Using a qualitative approach, this study revealed the perceptions of program implementers/targets during IHHP interventions. This is an acceptable method in complex and context-dependent community-based programs.

### Setting, sample and procedures

This study was conducted in intervention areas. Initially, a process evaluation committee (PEC) consisting of an independent team of researchers was assembled. The PEC members were five health professionals with previous experience in qualitative research. They became familiar with IHHP goals, objectives, strategies and interventional projects through 8 meetings in the presence of PEC members and IHHP board of directors (and some key stakeholders and program designers). The study participants consisted of key informants and others who had some experience with topics of interest to the targeted population, and included policy makers, stakeholders, projects administrators, local leaders, the community members etc. They were purposefully selected on the basis of their role in the interventional projects and information about the program areas, as well as recommendations from those already interviewed. A total of 60 participants were included in the study. Subsequently, feedback gained from the initial meetings was used to make decisions about the study's leading questions.

### Data collection and Interviews

Data collection involved in-depth semi-structured interviews and obtaining field notes during completion of the research over 3 years (2004-2006). This provided a source of formative data with which to identify issues and experiences gained during the implementation stage of the IHHP. Ten open-ended questions were used (Table 2). These questions were reviewed through meetings in terms of content, format, and audience appropriateness; subsequently they were modified to suit our participants and their specific roles.

**Table 2:** Qualitative interview guiding questions.

No	Open-ended questions
1	When the program has been implemented at this place, what factors do you think have affected the effective use of the interventions of this project?

2	Do you think it is better to change the way that work is done?

3	In your view, how successful was this intervention?

4	Do you think this intervention must be continued, modified, or discontinued? State your reasons.

5	Have the goals of your respective intervention been realized in the current system of your work?

6	What are some of the barriers or difficulties that you are encountering with the program? and which challenges are you confronting during the implementation of interventions?

7	Which kind of facilities/barriers are you confronting during the implementation of interventions?

8	State the degree of your satisfaction with this intervention.

9	How do you assess the future continuity of habits encouraged by the designed intervention?

10	Do you think this intervention can be implemented at the national level? Please state your reasons

Isfahan Healthy Heart Program, Iran, 1999-2000.

Two members of the PEC accompanied by their assistants conducted the interviews. They were provided with training to administer qualitative interviews on a one-on-one basis. Two pilot interviews were completed, transcribed verbatim, and reviewed by all of the members of the PEC to ensure that domains of interest were being fully explored. Then the interviewers were introduced to the IHHP managers and participants by sending letters explaining the purpose of the study; permission to collect data was obtained by use of forms. The investigators met the selected participants and then conducted face-to-face interviews. Each interview took approximately 1 hour to complete. Interviewers explored the questions shown in Table 2. All interviews were undertaken in an agreed private place (often a workplace). The interviews continued until reaching a level of information saturation. About six participants from each of the ten IHHP interventional projects were included in the study. Data collection and analysis were conducted simultaneously, and ceased when no new themes could be extracted from the interviews. The interviews were audio-taped with the participants' consent. The interviewer made detailed notes only in one instance where an interviewee preferred not to be audio-recorded. Taped interviews were transcribed verbatim very soon after the interviews.

### Research ethics

The ICRC Ethics Committee (NIH Code: FWA 0000t8578) approved the study. Informed consent was obtained from the participants prior to interviews; the participants were ensured about confidentiality and anonymity. All data were protected and were made available only to the authors; tapes of interviews were kept in a locked drawer.

### Analysis

Collection and analysis of qualitative data were done simultaneously. A modified thematic analysis was used to analyze the data obtained from the transcripts, as well as relevant field notes; it was used to identify explicit and implicit themes, and essences or patterns within the text in relation to the study aims and objectives [10]. Transcripts were independently read by the two principal researchers (M.A & K.R), who subsequently coded, identified and categorized the existing themes. Another researcher (H.A) assessed the work on a subset of transcripts to ensure consistency. The analysis process was repetitive, the texts were coded and common codes were combined to identify related themes [11]. After analyzing each interview (and related notes), sections of texts coded to a particular theme were examined across the datasets and then the overall themes were developed [12]. We ensured that any emergent themes and concepts were developed from the data and not from a predetermined hypothesis. Theme choices were collectively discussed and the final interpretation was produced and agreed upon. Table 3 shows the list of themes and code definitions.

**Table 3:** Themes, sub-themes and their definitions/descriptions.

Theme	Sub-theme	Definition/description	Narration examples
Experiences with resources	Resources (human work force and hardware) were perceived as key factors in carrying out the projects. They were both *facilitating *and *hindering*		*hindering:* Participant (no. 6-HLCP): "The major problem is insufficient number of nurses in the hospitals, unfortunately." Participant (no.2-HLCP): "We can't follow up the implementation the interventions, because we have few professionals against high duty load." *facilitating *: Participant (no.6-HLCP): "We prepare slides as powerpoints; get pamphlets to nurses who agreed to become trained." Participant (no.5-HLCP): "All instruments and resources were prepared by the hospital managerial team and nobody had helped them." Participant (no.1-HLCP): "I brought my personal computer from my own shop and installed educational materials on it."

Attitude toward the program	It implies any *positive or negative beliefs (barriers) *about IHHP, in terms of program effectiveness and feasibility, directors' qualifications etc.		*positive believes:* Participant (no. 6-HLCP): "Nursing supervisors in hospitals are very concerned... we printed a folder on some topics for patients' education.... It seems to be successful. Many physicians requested these educational materials for their private offices." *negative believes:* Participant (no. 5-WIP): "Initially, there was some resistance on the part of workers, employees, cooks, etc. Yes, resistance persists actually" Participant (no. 2-WIP): "Among the barriers were the economic concerns of some managers at work which was obvious. Any plan, like regular daily exercise for employees, that took up some of the work time, however little, would bring about resistance"

Enabling factors	Effective education	Applicable and understandable education had encouraged learning and adherence to the program	Participant (no. 6-HPEP): "I myself, enjoyed the subject of the tobacco control program" Participant (no. 6-HLCP): "Patients need a holistic approach. For example they might ask: When am I allowed to drive? ... Some topics in our classes cannot answer the patients' questions."
	
	Positive reinforcement	Offering positive feedback to people increased acceptance and continuation of desirable behaviors/healthy life-style	Participant (no. 1 - WIP): "We have an antismoking program named Quit and Win. It was preformed regularly." Participant (no. 3 - WIP): "We planned some competitions like caricatures, among employees and their families to encourage smoking cessation. Consequently, all their family members were persuaded to participate."
	
	Creative activities	Some activities which have increased stakeholders and other participants' incentives and adherence to the program	Participant (no.1 - HLCP): "We set up stations and an exhibition that introduced IHHP pamphlets. In fact these activities drew people's attention to communicate with us."
	
	Recognition and appreciation	It means to acknowledge that the peoples' compliance has enhanced their participation in the program interventions	Participant (no. 1 - HPEP): "Now, education is valuable, it affects employee's evaluation and job improvement. Thus employees are attracted to education." Participant (no. 8 - HPEP): "Nurses don't motivate, because they have lost their professional values. There is no difference between active and passive employees."
	
	Sensitization	Alerting and informing the people against detrimental effects of risky behaviors has encouraged them to adopt healthy behaviors	Participant (no. 2 - HLCP): "The audiences in the seminars understood how useful the secondary prevention and rehabilitation training programs are for patients." Participant (no. 3 - WIP): "After recent interventions in our factory, if the employees don't get vegetables and dairy diet during a week, they will complain about it to the factory health center."

Isfahan Healthy Heart Program, Iran, 1999-2000.

HPEP: Health Professionals Education Project

WIP: Worksite Intervention Project

HLCP: Healthy lifestyle for Cardiovascular Patients

### Quality assurance of data analysis and interpretation

The reliability and validity of the study was strengthened by including adequate participants, conducting the analytical process actively, and reaching a level of saturation. Relevance of these strategies has been supported [13]. Audio recordings and verbal transcripts were used to collect data. Text coding and data analysis were performed independently by three senior researchers and the results were discussed by the PEC members to identify any inconsistencies. Additional strategies were adopted to strengthen the study; these included investigator triangulation and peer-checking of the themes [14].

## Results

There were several interventions in each project, hence a variety of themes and sub-themes emerged from this study. IHHP was performed in multiple settings (Annex 1), however, the main target groups who underwent interventions were the general population, health professional and cardiovascular patients. Here we present the results obtained from the HLCP, HPEP and WIP projects. Most of the project managers were selected from amongst policymakers, stockholders and academics. Hence, most of the participants in the current quantitative research had positive attitudes to the planned interventions, which resulted in higher commitment to the program. They felt more responsible and adjusted their performance using feedbacks. Data emerging from this qualitative study produced several themes and related sub-themes, as follows:

### Resources

Accessibility of resources was emphasized by the participants; it affected the implementation of interventions. Adequate and appropriate allocation of resources yielded satisfactory results in line with program goals. Lack or shortage of resources, limitations in material and human resources, increased personnel responsibility, and changes in management, led to serious problems. Availability of human resources critically affected the implementation of IHHP interventions. The participants said they faced considerable shortage of human resources. The IHHP board of directors had relied on the participants themselves to appropriately respond to this challenge, i.e. the program aimed to make the participants self-enterprising, for instance, many of the educational materials prepared in IHHP projects were used by nurses for peer education; here the resource was not supplied directly by IHHP directors, rather by program stakeholders and the community. This reflects improvement of culture in a community-oriented health program. Educational technology was found to be of great importance second to human resources.

### Attitudes to the program

We defined 'attitude' as any positive or negative belief about the program's effectiveness, feasibility and rationale, as well as feelings about program designers/directors. We found from the participants' experiences that positive attitudes, especially those of project managers strengthened their commitment to the interventions, enabling them to achieve a higher level of program acceptance. Another finding related to the lack of awareness of program goals, and negative attitude to program effectiveness among some health professionals/policy makers. Project managers spent much time and energy solving this conflict. Our analysis suggests that a key barrier to implementation was the stakeholders' negative attitude to IHHP.

### Enabling factors

Project managers arranged meetings with stakeholders to draw their compliance with planned IHHP activities. Based on the participants' experiences, some of these activities were helpful and were accepted by the stakeholders as a "pleasant experience". The factors perceived as being "enabling" are:

#### Effective education

The trainees were pleased with their education program and found it in line with their occupational needs.

#### Positive reinforcement

Program planners found more incentives to encourage the acceptance/continuation of desirable behaviors. For example, one method used to encourage smoking cessation was to establish a reward system.

#### Creative activities

We indentified activities within the programs for prevention, treatment and rehabilitation of cardiovascular disease which increased motivation and enhanced the stakeholder's push toward program goals.

#### Recognition and appreciation

Gaining appreciation was a great challenge in implementing interventions, especially in hospitals. Behaviors related to program objectives need not only recognition, but also appreciation.

#### Sensitization

It attempts to build an acceptable atmosphere where the stakeholders learn the educational concepts underlying IHHP. We found that improving people's knowledge improved their behaviors.

#### Consistent participation

The implementation of interventions requires advocacy and cooperation of other organizations. Communication with stakeholders is essential to the continuum of interventions. Participation included formal and/or informal relations to the program's multidisciplinary background. In addition, the quality of relations predicted agreeable outcomes.

## Discussion

Our findings supported the notion expressed as "more staff, more training, more skills and strengthened health systems" [15]; financial, technological and human resources etc were central to achieving IHHP goals and objectives. Although IHHP aimed to draw upon a spontaneous motion toward healthy lifestyle, our findings stress the importance of timely and adequate provision of resources for the initiation and continuation of the program. All interventional projects tend to suffer from resource scarcity. The importance of resource management [16,17] and the role of health care as a dynamic sector [18] has been demonstrated. Development of appropriate approaches for analyzing the need for health/social care services and resources is important [19]. We found that a responsive atmosphere aided by creative activity-planning, providing positive feedback for healthy behaviors, and developing materials tailored to the stakeholders' needs facilitated implementation of interventions and helped overcome some practical limitations; a fertile atmosphere receptive to new programs was conceived. In order to direct human resources toward change, it is necessary to consider three relevant elements, namely attitude, expertise and skill [20]. Attitudes toward partnership in the program, as well as perceptions about the usefulness of the planned interventions and their outcomes constituted an important concern in the program. As the attitudes and values of individuals are the main factors for promoting positive behaviors and transforming them into opportunities for success [20], it is important to assess the participants' attitudes toward change [21].

Based on our participants' experiences, enabling factors were the most interesting ones. We found that readiness of stakeholders and the community to accept new changes was considerably grounded in their motivation. The various educational sessions drawn up by program designers were part of a core strategy to achieve this goal. To make these sessions more effective, social, environment and contextual factors were taken into account. The participants in IHHP i.e. program planners, stakeholders, and the community joined in a collaborative approach of both intra- and inter-sectoral nature. Some of the problems faced in the process were caused by traditional, professional, and organizational values and/or interpersonal incompatibilities. The emphasis on collaboration is consistent with the efforts encouraging a collaborative approach to health services delivery [22]. This is especially true with respect to self-managed work teams, applicable to community-based programs [22]. The results also stress the importance of administrative formalization initiatives to enhance collaboration among professional/organizational groups. The relevance of such view is well documented. Formalization of functions and processes appear to be an interesting means to further collaboration [22].

In summary, most of the participants expressed satisfaction with the program. This may have been due to IHHP's integration with existing systems/facilities at the community level. The PEC found that IHHP designers had developed strategies to provide accessible and useful services to all users. Notably, they could overcome resource shortages through appropriate management. They employed various strategies, such as training and information programs to assess the special needs/opinions of target groups, risk sensitization programs, inter-sectoral collaboration, alliance formation, and behavioral change through inductive approaches etc; this way, they tackled some of the negative attitudes to practicality/usefulness of program.

## Conclusion

This study highlights the important role of resource availability, as well as facilitating/hindering factors. Acceptance of interventions and compliance were determined by attitudes/perceptions about the feasibility/effectiveness of the program. Face-to-face meetings and distribution of educational materials in the community reduce resistance to the program.

Organized planning and consistent participation are essential to success of interventions and adherence to the program. Successful strategies and interventions were officially integrated into the health system. The findings and recommendations of evaluation were used in subsequent program planning. We propose that simple IHHP-type interventions be adapted for implementation and integration in communities with limited financial and/or human resources.

## Competing interests

The authors declare that they have no competing interests.

## Authors' contributions

NS: director of the project and design the study and rewrote the article to its present form. KR: coordinated the project and responded to the comments of the referees. MA: drafted the original version of the manuscript. HAA: performed the qualitative analysis and commented on the qualitative design of the study. SZ: help to coordinate of the project. All authors have read and approved the final manuscript.
